# An Unusual Presentation of Pyloric Stenosis: A Case Report

**DOI:** 10.7759/cureus.40578

**Published:** 2023-06-17

**Authors:** Shreya Sodhani, Aashka H Patel, Yesenia Morales

**Affiliations:** 1 Pediatrics, The Brooklyn Hospital Center, New York, USA; 2 Neonatology, The Brooklyn Hospital Center, New York, USA

**Keywords:** gastrointestinal obstruction, non- bilious vomiting, abdominal distension, newborn, vomiting, atypical presentation, pyloric stenosis

## Abstract

A full-term newborn female presented with non-bilious emesis immediately after feeding and abdominal distension on day one of life with neither palpable abdominal mass nor electrolyte derangements. The baby was initially admitted to rule out gastrointestinal obstruction versus sepsis as a cause of vomiting and abdominal distension. Initial imaging studies involving an upper gastrointestinal (GI) series showed obstruction at the level of the duodenum, but it was only during surgical exploration that the diagnosis of infantile hypertrophic pyloric stenosis was made. This case report highlights the atypical presentation of pyloric stenosis and the need to investigate cases of vomiting immediately after feeding in a newborn with ultrasonography at the least to minimize complications.

## Introduction

Vomiting in neonates can result from a wide range of causes, including surgical as well as nonsurgical, such as sepsis, metabolic derangements, infectious causes, and obstructions within the gastrointestinal (GI) tract. Pyloric stenosis typically presents with postprandial projectile, non-bilious vomiting, hypokalemic and hypochloremic metabolic alkalosis, dehydration, and weight loss [1]. We present a case that illustrates an atypical presentation of pyloric stenosis and emphasize the need to consider it as a cause of vomiting in a newborn who falls outside the typical age of presentation, which is three weeks to three months [2].

## Case presentation

**Figure 1:**
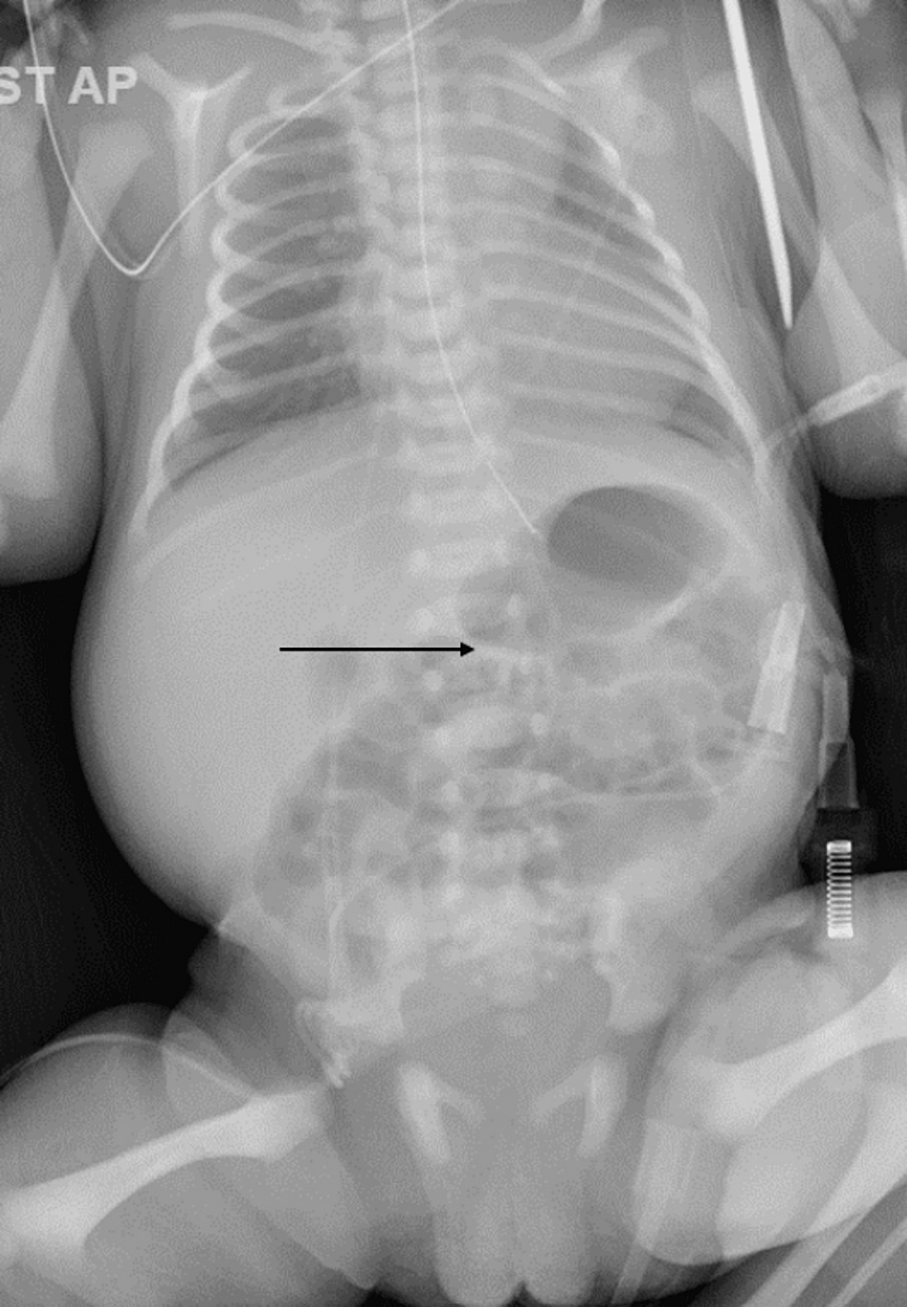
Abdominal X-ray with normal bowel gas pattern and no dilated bowel loops visualized (arrow)

The patient was a full-term female born at 37+0 weeks of gestation via elective C-section to a 41-year-old mother para 1001. The pregnancy had been complicated by advanced maternal age, obesity, and chronic hypertension. However, the delivery had been uncomplicated and the baby had been sent to the newborn nursery for routine care. The baby’s birth weight was 3140 g and she had a normal physical exam and stable vital signs. At six hours of life, the baby was noted to have non-bloody, non-bilious emesis, and the subsequent exam was significant for abdominal distension. An orogastric catheter was inserted with the aspiration of milk and air, resulting in mild improvement in distension. On further exam, normal bowel sounds were heard in all quadrants and there was no palpable mass. The baby was admitted to the NICU due to the suspicion of early-onset sepsis as a likely cause of this vomiting and abdominal distention. CBC and electrolytes were unremarkable. Empiric antibiotics were started pending blood culture results. The baby passed urine and stool within 24 hours of life. Abdominal X-ray on admission showed a normal gas pattern with no other obvious abnormalities (Figure 1).

**Figure 2:**
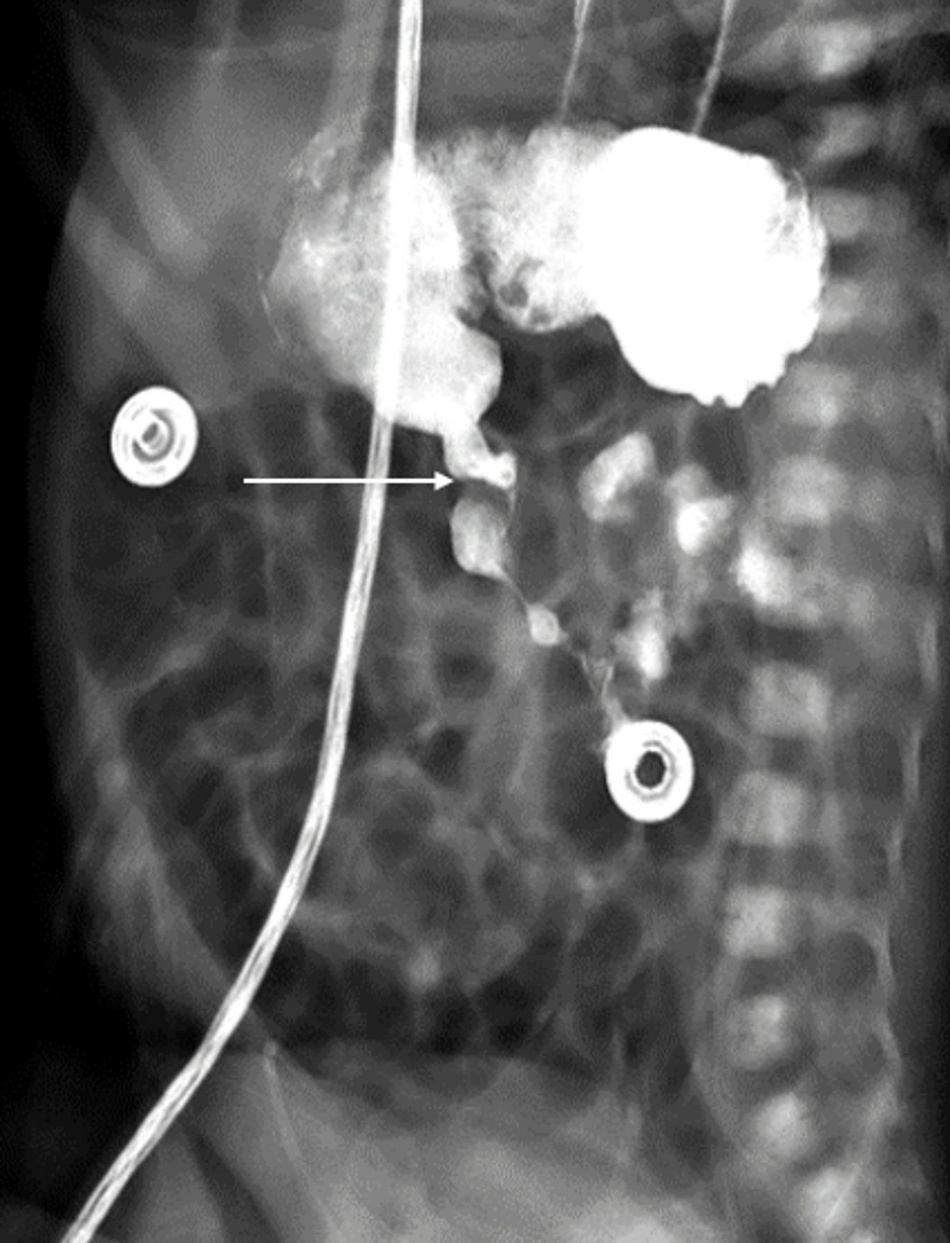
Upper gastrointestinal series showing contrast passing through the pylorus (arrow)

With progressive feeds of just 10 ml, the baby continued to have non-bilious emesis and progressive distension, raising concerns for proximal bowel obstruction. The physical exam showed unchanged results. By day two of life, the blood culture showed no growth, and hence antibiotics were discontinued after ruling out sepsis. Due to continued feeding intolerance, an upper GI series was performed on day three, which showed contrast passing through the pylorus, mild dilation of stomach and bowel loops as well as narrowing of the second part of the duodenum (Figure 2).

**Figure 3:**
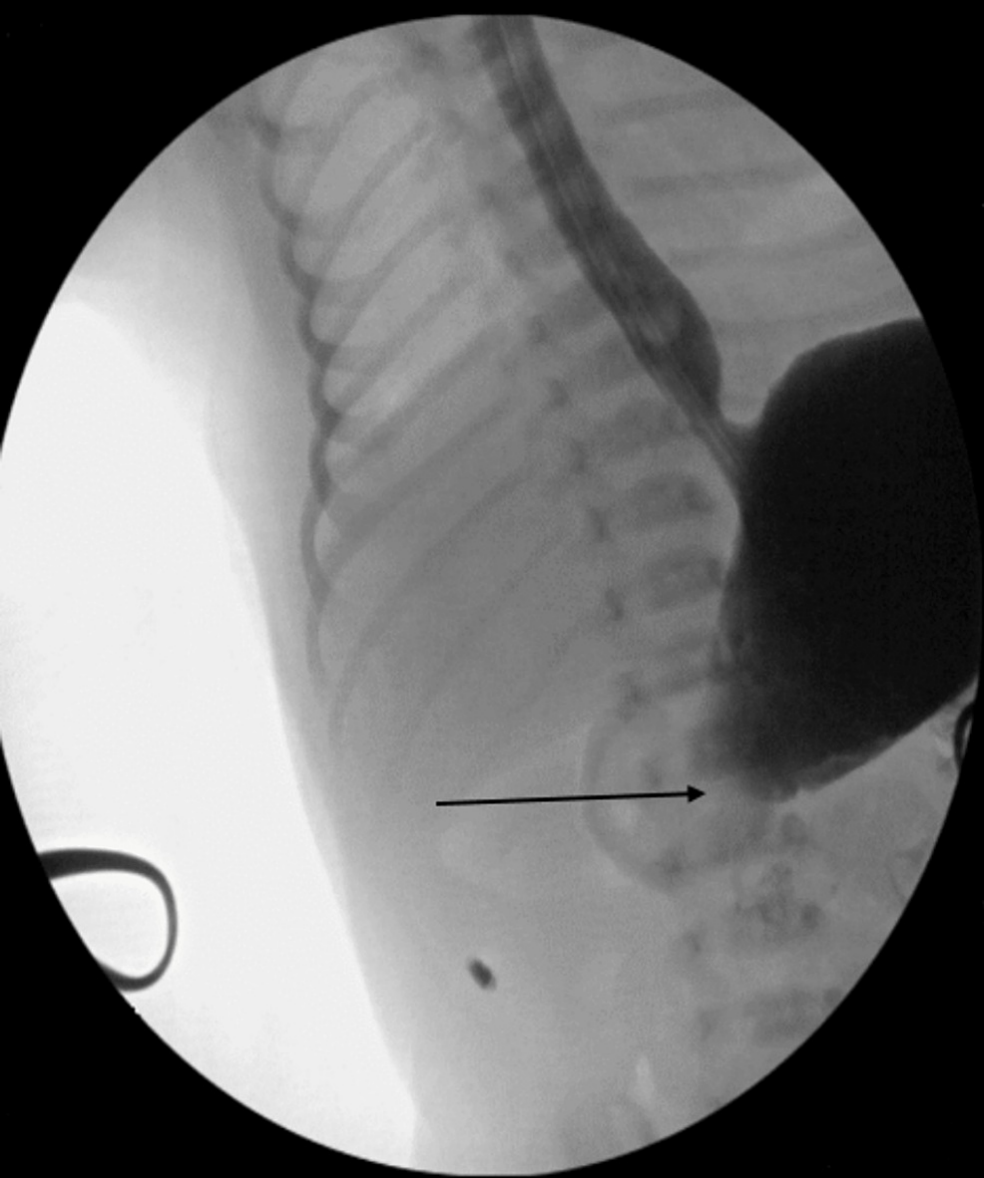
Intraoperative contrast study showing contrast stopping around the pylorus (arrow)

The surgical team was consulted for possible annular pancreas or duodenal web. At this point, it was decided to perform an exploratory laparotomy. During surgery, firmness was palpated in the pylorus. An intraoperative contrast study was performed and the contrast appeared to stop around the pylorus (Figure 3). A rubber drain was placed distally through a gastrostomy and contrast was pushed distal to the pylorus, which moved all the way through the small bowel. At this point, a diagnosis of pyloric stenosis was made.

A pyloromyotomy was performed without any complications. Incremental oral feedings were tolerated well postoperatively. Before discharge, the patient was taking 45-50 ml of expressed breast milk orally without any further episodes of emesis or abdominal distention.

## Discussion

Pyloric stenosis is a common cause of infantile GI obstruction, occurring in 2-3.5 per 1000 live births [3]. A majority of them present between three weeks and three months after birth [4]. Infants rarely present before two weeks of age, and those who present early are more likely to have a positive family history of pyloric stenosis [5]. Typically, those affected are premature, firstborn, and mostly males, in whom the disease is four to six times more common than females [6-8]. Other typical risk factors include a positive parental history of pyloric stenosis, maternal smoking, and early exposure to erythromycin (<2 weeks of age) [4,9,10]. Our patient was a full-term, second-born, female child who showed symptoms within the first week of her life. She had a negative parental history of pyloric stenosis and there was no history of maternal smoking or early macrolide exposure.

Early presentation of pyloric stenosis, occurring during the first seven days of life, makes the diagnosis very challenging. In day-to-day practice, vomiting in a newborn most often suggests overfeeding, poor positioning during feeds, or regurgitation. However, it is imperative to consider more serious conditions like sepsis, necrotizing enterocolitis, inborn errors of metabolism, or obstructive lesions of the GI tract. These conditions may be less common but they pose a greater threat if not promptly identified and treated.

Pyloric stenosis typically presents with projectile non-bilious vomiting, usually occurring immediately after feeding, and after vomiting, the infant appears hungry and wants to be fed again (hungry spitter). A pyloric mass, which is described as olive-shaped, can be palpated in the right upper quadrant and gastric peristaltic waves are often visible just prior to vomiting. When symptoms are prolonged, vomiting may result in hypokalemic and hypochloremic metabolic alkalosis [11]. Also, other unusual aspects of our case were the absence of the projectile nature of vomiting, palpable mass on the exam, and visible peristalsis after test feedings. Since the patient continued to be symptomatic with some indication of obstruction albeit inconclusive, the decision was made to proceed with surgical exploration. During surgical exploration, it was found that the patient had firmness in the pyloric region, after which the diagnosis of early pyloric stenosis was made. 

Conventionally, the modality of choice for the diagnosis of pyloric stenosis is an abdominal ultrasound, which shows pyloric length >18 mm or pyloric muscle thickness >4 mm [12]. A sonographic evaluation may have helped with earlier diagnosis, but no established guidelines exist for infants in this age group. Immediate treatment involves the correction of dehydration and metabolic disturbances, followed by surgical correction of the stenosis.

## Conclusions

This case report highlights the need for a higher index of suspicion for pyloric stenosis despite the absence of “typical risk factors.” Our objective was to emphasize the need to consider pyloric stenosis as a possible differential when encountering infants with vomiting in the first two weeks of life. There should be a low threshold for using ultrasound in such cases as it is the diagnostic method of choice and is a cheap, readily available imaging option with no radiation exposure.
